# Allyl Dimethyl Sulfonium: A Novel Urinary Biomarker of *Allium* Consumption

**DOI:** 10.1021/acs.jafc.5c01077

**Published:** 2025-04-02

**Authors:** Lorenz Steiner, Andrea Raab, Joerg Feldmann, Walter Goessler, Bassam Lajin

**Affiliations:** 1 Institute of Chemistry, Analytical Chemistry for Health and Environment, University of Graz, Universitaetsplatz 1, Graz 8010, Austria; 2 Institute of Chemistry, TESLA, University of Graz, Universitaetsplatz 1, Graz 8010, Austria; 3 Institute of Chemistry, ChromICP, University of Graz, Universitaetsplatz 1, Graz 8010, Austria

**Keywords:** allyl methyl sulfide, allicin, garlic, metabolism, urinary excretion, biomarkers of food intake (BFI)

## Abstract

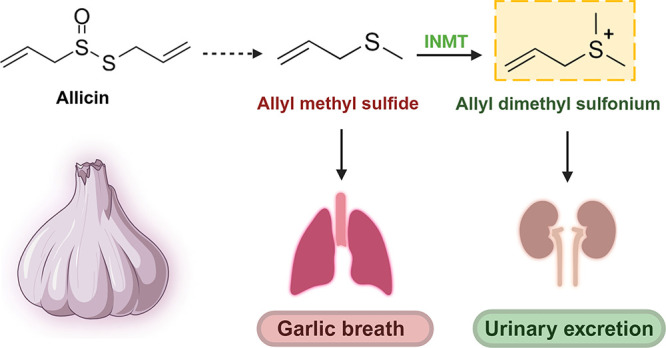

Allyl methyl sulfide (AMS) is an odorous and bioactive major metabolite produced following *Allium* food consumption and is regarded as the culprit behind the “garlic breath”. Indoleethylamine *N*-methyltransferase (INMT) can methylate a variety of thioethers to their respective sulfonium ions in humans, aiding in their urinary excretion. We hypothesize that AMS may serve as a novel target for INMT and be metabolized to the allyl dimethyl sulfonium (ADMS) ion, which would constitute a previously undescribed pathway for metabolism of *Allium* food. ADMS was synthesized, and analytical methods were developed to explore its existence and characterize its levels in humans. ADMS was indeed consistently detected in all volunteers over 6 weeks without dietary intervention and found to strongly respond to controlled garlic supplementation. Striking interindividual variability in urinary ADMS was observed and found to mirror other products of INMT, which is attributable to genetic variation. ADMS is a novel metabolite in humans, and its remarkably variable production suggests variable response in body odors and health effects of *Allium* food and can be used to assess *Allium* consumption in the general human population in future epidemiological studies. More products of INMT that can serve as biomarkers of sulfur-rich food intake await discovery.

## 1 Introduction

Plant species of the genus *Allium* are rich sources of bioactive organosulfur compounds.^1−3^*Allium sativum* (garlic) and *Allium cepa* (onions) are the most commonly consumed *Allium* vegetables by humans, and their nutritional benefits have been a subject of numerous studies.^4,5^

Alliin is a major compound in garlic with concentrations reported at 0.6–1.4% w/w (fresh weight basis).^6^ Mechanical disruption of garlic releases the enzyme alliinase from cellular compartments converting alliin to various thiosulfinates, the major compound of which is allicin (0.3–0.5% w/w),^6^ which has been the focal point in investigating the health effects of *Allium* species.^7−9^ However, these thiosulfinates, including allicin, are unstable and quickly degrade to multiple organosulfur compounds, particularly diallyl disulfide (DADS) and diallyl trisulfide (DATS), which together constitute ca. 73% w/w of garlic essential oil.^10^ Following garlic consumption, these compounds can be reduced by glutathione to allyl mercaptan, which is methylated to allyl methyl sulfide (AMS), a major odorous garlic-derived sulfur metabolite in humans, which is excreted via exhalation and considered the main culprit for what is commonly referred to as the “garlic breath”.^11,12^

In 1988, Mozier et at.^13^ isolated a mammalian enzyme from a mouse liver and demonstrated through *in vitro* tests that this enzyme is capable of methylating a variety of compounds to their respective “-onium” derivatives. Notable example precursors that were tested are dimethylsulfide, dimethylselenide, and diallyl sulfide, which were found to be methylated to trimethylsulfonium (TMS), trimethylselenonium (TMSe), and diallyl methyl sulfonium, respectively.^13^ This enzyme, which was referred to as thioether *S*-methyltransferase, is expressed in humans^14^ and is also known to be capable of methylating tryptamine to methyltryptamine and dimethyltryptamine, which are involved in neurotransmission.^15^ Due to the latter *N*-methylation activity, the enzyme is also referred to as indolethylamine *N*-methyl transferase (INMT).

AMS and its aforementioned precursors are hydrophobic volatile compounds and, therefore, can accumulate in the human body in the absence of an effective excretion pathway. AMS is a bioactive metabolite, which is difficult to measure due to its volatility, and there is a need for novel and easily measurable urinary biomarkers that reflect the long-term consumption of *Allium* food, which would aid in investigating effects on human health serving as a more reliable alternative to food questionnaires.

Although Mozier et al.^13^ reported the methylation of a range of thioethers, allyl methyl sulfide (AMS) was not tested. However, the structural similarity of this common and biologically active garlic-derived human metabolite to dimethyl sulfide and diallyl sulfide suggests that allyl dimethyl sulfonium (ADMS) can be produced by humans; however, this has not been previously investigated and the existence and levels of ADMS in humans are unknown. Therefore, in the present study, we hypothesize that AMS may serve as a precursor for INMT, yielding the allyl dimethyl sulfonium (ADMS) ion, which would be readily excreted in human urine to aid in clearance of AMS and serve as a novel pathway for the metabolism of *Allium*-derived organosulfur compounds in humans.

## 2 Materials and Methods

ADMS was synthesized by dissolving dimethyl sulfide (620 mg) and allylic alcohol (290 mg) in dry dichloromethane (5 mL). At −10 °C, a tetrafluoroboric acid diethyl ether complex (810 mg) was added, and the mixture was stirred at room temperature for 4 days. After residual volatiles were removed in vacuo, the product was obtained as an oil (910 mg, 95% yield). For the isotopically labeled internal standard (ADMS-*d*_6_), a similar procedure was applied except for using isotopically labeled dimethyl sulfide (DMS-*d*_6_, Sigma-Aldrich, Germany, purity 99 atom % D). A standard solution of 50 nM ADMS-*d*_6_ was prepared in water and coinjected with samples and standards (see below).

An analytical method was developed for the identification and quantification of ADMS in urine based on high-performance liquid chromatography coupled with electrospray ionization tandem mass spectrometry (HPLC-ESIMS/MS). An Agilent 1260 Infinity II LC system (Agilent Technologies, Waldbronn, Germany), equipped with a quaternary 1260 Infinity II Flexible Pump (G7104C), a multisampler (G7167A), and a Column Thermostat (G7116A), was coupled with a triple quadrupole Ultivo LC/TQ system (G6465B, Agilent Technologies, Waldbronn, Germany). Separation was performed on a Zorbax Eclipse Plus C18 RRHD column (50 × 2.1 mm, 1.8 μm particle size, Agilent Technologies, Waldbronn, Germany) with a mobile phase containing heptafluorobutyric acid as an ion-pairing reagent. Urine was injected without preparation except for filtration through 13 mm Nylon-66 syringe filters (pore size 0.22 μm BGB Analytik GmbH, Germany). An isotopically labeled internal standard (ADMS-*d*_6_) was used to account for urine matrix effects, and two mass transitions were used to ensure the selectivity of detection. Table 1 lists detailed chromatographic and mass spectrometric conditions. To confirm identification, high-resolution mass spectrometric measurements using an Agilent 6546 quadrupole time-of-flight mass spectrometer (Agilent Technologies, Waldbronn, Germany) were performed under experimental conditions similar to those listed in Table 1 except for substituting the ion-pairing reagent with a cation exchange PRP-X200 chromatographic column (Hamilton, USA), see Figure S1. Trimethylsulfonium (TMS) and trimethylselenonium (TMSe) were determined according to the methods previously described.^16,17^

**Table 1:** Instrumental Conditions for the Determination of Allyl Dimethyl Sulfonium (ADMS) in Human Urine

**HPLC**	
Stationary phase	Zorbax Eclipse Plus C18 2.1 × 50 mm, 1.8 μm
Mobile phase	A: water B: 1.0% (v/v) heptafluorobutyric acid + 0.1% acetic acid, pH adjusted to 4.5 with ammonia C: 1.0% acetic acid, pH adjusted to 4.5 with ammonia D: methanol isocratic elution: 20% B + 15% C + 8% D
Column temperature (°C)	35
Flow rate (mL min^–1^)	0.3
Injection volume (μL)	1.0 μL urine + 0.5 μL ISTD (ADMS-*d*_6_), 50 nM
**ESIMS/MS**	
Nebulizer gas temperature (°C)	350
Nebulizer gas flow (L min^–1^)	10
Nebulizer pressure (psi)	35
Sheath gas (°C)	400
Sheath gas (L min^–1^)	12
Fragmentor (V)	50
Collision energy (V)	5 (quantifier, ISTD), 15 (qualifier)
Capillary voltage (V)	+2500
Monitored transitions	103 → 61 (quantifier) 103 → 41 (qualifier) 109 → 66 (ISTD)

To characterize the urinary concentrations of ADMS, a total of 53 urine samples were included in the study. The study population and sample collection procedure were previously described.^18^ In brief, eight volunteers (age range: 18–60, age mean ± SD: 37 ± 13 years, 5 females, and 3 males) living in the city of Graz were recruited. Volunteers donated one morning urine sample (first-pass) in 300 mL Corning sample collection containers on a weekly basis over six consecutive weeks. The volunteers were following a local western diet with occasional use of *Allium* vegetables (garlic and onions) as cooking ingredients, and no dietary intervention was made except for follow-up experiments where selected volunteers were asked to increase dietary garlic consumption to 10–30 g/week over 4 weeks. Informed consent was obtained from the participants, the study was performed in compliance with the Declaration of Helsinki, and urine collection was approved by the ethical committee at the University of Graz (GZ: 39/46/63).

## 3 Results and Discussion

**Figure 1:**
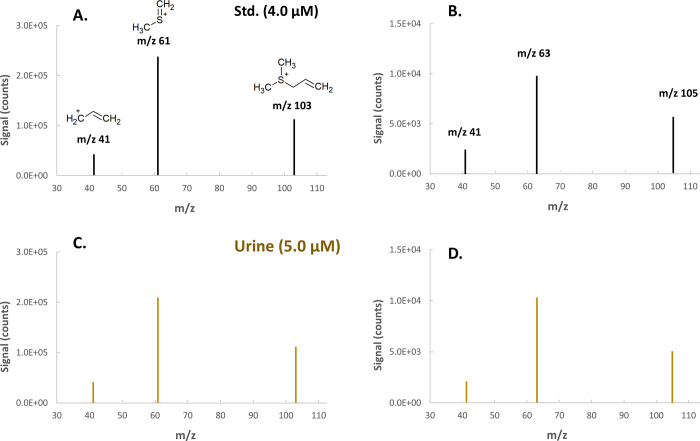
MS/MS spectra recorded with a product ion scan using the nominal masses corresponding to the two sulfur isotopes in allyl dimethyl sulfonium (*m*/*z* 103 and 105) as precursor ions. The spectra in pure standard (A, B) and in urine (C, D) are shown.

**Figure 2:**
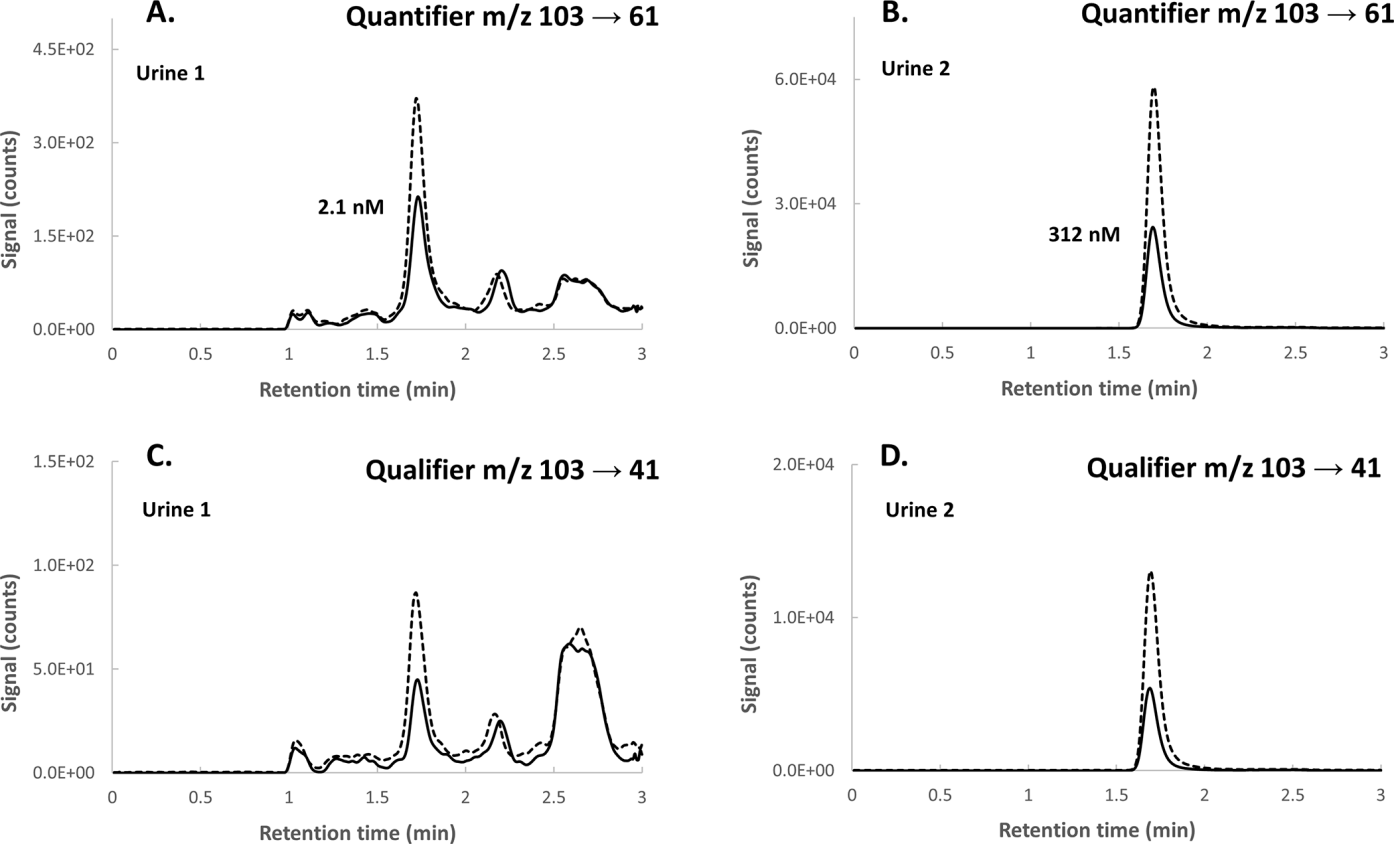
Detection of allyl dimethyl sulfonium in urine based on the quantifier (A, B) and qualifier (C, D) mass transitions in urine samples with low (urine 1) and high (urine 2) native concentrations. The solid lines show the chromatograms of the native urine, and the dashed lines show spiked urine at 2 and 500 nM for urine 1 (A, C) and urine 2 (B, D), respectively.

Identification of ADMS in human urine was based on (i) matching of the product ion MS/MS spectra of ADMS at *m*/*z* 103 and 105, which correspond to two sulfur isotopes (^32^S and ^34^S), between in-house synthesized standard and urine (Figure 1), (ii) chromatographic coelution experiments based on multiple mass transitions (103 → 61 for the quantifier transition and 103 → 41 for the qualifier) at low (2.0 nM) and high (300 nM) endogenous urinary ADMS concentrations (Figure 2), and (iii) exact mass measurements with high-resolution mass spectrometry (QTOF) with a theoretical calculated mass of 103.058 and measured mass of 103.057 (mass accuracy ± 5 ppm) (Figure S1).

The developed analytical method used for quantification in a total of 53 screened urine samples was based on liquid chromatography coupled with electrospray ionization tandem mass spectrometry (HPLC–ESIMS/MS) and involved monitoring the target compound with two mass transitions (qualifier and quantifier) to ensure selectivity of detection in the complex urine matrix. An isotopically labeled internal standard was employed (ADMS-*d*_6_) to enable direct injection of untreated urine while accounting for matrix suppression effects and ensuring accurate quantification. The limit of detection (LOD) for ADMS based on the urine matrix was 0.2 nM. The analytical method was evaluated for repeatability (RSD% < 10%), recovery (85–110%), and linearity (linear range = 0.5 – 100 nM, *r*^2^ = 0.9999).

**Figure 3:**
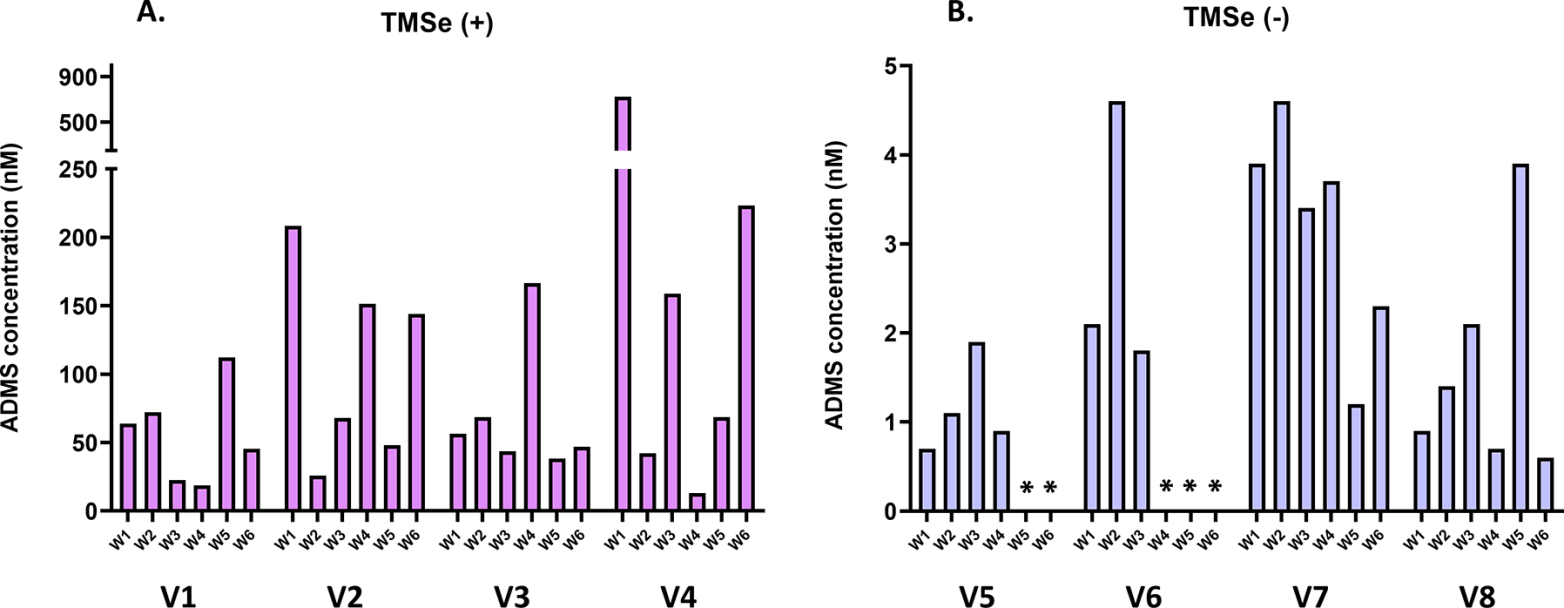
Investigating the inter- and intraindividual variability in the urinary excretion of allyl dimethyl sulfonium. The graph shows concentrations in urine collected weekly over six consecutive weeks (W1–W6). All concentrations were adjusted to specific gravity. The volunteers (V1–V8) were grouped according to their TMSe status to TMSe producers (+) (A) and non-producers (−) (B); for details, see the text. Asterisks indicate concentrations below LOD (0.2 nM).

**Figure 4:**
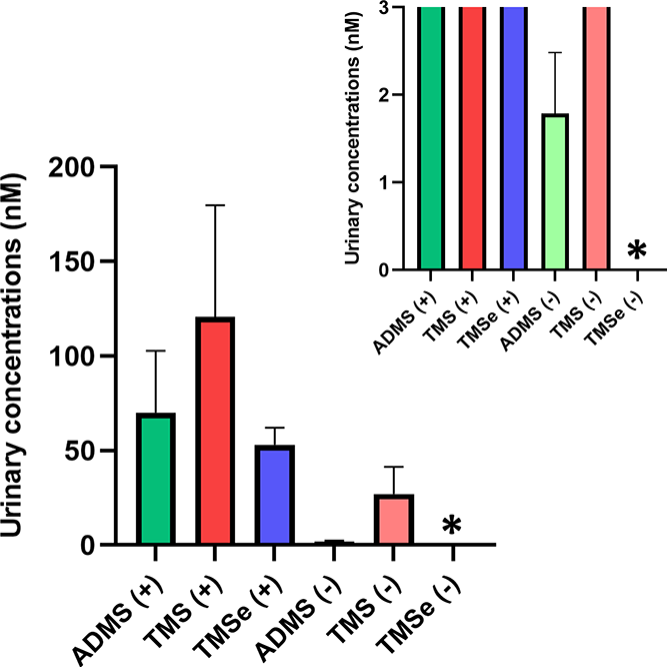
Impact of the TMSe production phenotype on urinary allyl dimethyl sulfonium (ADMS) and trimethylsulfonium (TMS). Urinary concentrations were adjusted to specific gravity, and the weekly samples were grouped according to the TMSe status (+ for producers and – for non-producers). TMSe is nondetectable in TMSe non-producers (LOD 1.0 nM), denoted with an asterisk.

To investigate the consistency in ADMS occurrence in urine and its intraindividual variability, the volunteers were asked to collect urine repeatedly on a weekly basis for six consecutive weeks. The volunteers followed a normal western diet with moderate and occasional consumption of *Allium* plants (e.g., garlic and onions) as cooking ingredients. No dietary intervention or supplementation was involved in this part of the study. ADMS was consistently detected in urine from the studied volunteers and in the vast majority of urine samples (43 of 48 samples). The urinary levels were in the nanomolar range (Table 2), and the mean concentrations showed significant interindividual variability as two distinct clusters were observed (56–205 nM in volunteers 1–4 and 1.2–3.2 nM in volunteers 5–8) (Table 2). This large interindividual variability could not be explained by the diet. A similarly striking interindividual variability has been consistently observed over more than two decades for TMSe production, where humans are grouped into the so-called “TMSe producers” and “TMSe non-producers”,^19^ which are clearly distinguished by a >50-fold gap in urinary TMSe concentrations. This interindividual variability in TMSe production has been recently explained by single-nucleotide polymorphisms in the *INMT* gene,^20^ and we recently confirmed that these genetic polymorphisms are similarly associated with the production of the sulfur analogue TMS.^21^ It is therefore very likely that these genetic variants are largely responsible for the interindividual variability in urinary ADMS observed in the present study. This was made clear by grouping the volunteers in this study according to their TMSe production phenotype (Figure 3). However, the impact of this genetic variability appears to vary according to the INMT substrate, as it was considerably lower for TMS (5–10 fold) than for ADMS (25- to 50-fold) and TMSe (>50-fold) (Figure 4 and Table 2). Since AMS is an odorous metabolite and its emission from body through the skin and via exhalation is largely responsible for body odors following the consumption of garlic,^22^ the above-described large interindividual variability in the production of its urinary product ADMS has implications for susceptibility of humans to what is commonly referred to as the “garlic breath”.

**Table 2:** Measured Urinary Concentrations in the Studied Eight Volunteers^d^

	V1	V2	V3	V4	V5	V6	V7	V8
ADMS (nM)	47	85	61	100	1.1	2.6	2.9	1.3
	56 ± 35	108 ± 71	70 ± 48	205 ± 266	1.2 ± 0.5	2.8 ± 1.5	3.2 ± 1.2	1.6 ± 1.2
	19–112	25–208	38–167	13–724	<0.2–1.9^a^	<0.2–4.6	1.2–4.6	0.6–3.9
TMS (nM)	52	153	77	350	20	14	23	84
	62 ± 41	191 ± 112	86 ± 45	370 ± 113	21 ± 7	17 ± 10	28 ± 22	119 ± 76
	28–119	31–357	42–151	172–481	12–32	4.3–33	10–69	11–199
TMSe (nM)	70^b^	64	33	55	<1.0^c^	<1.0	<1.0	<1.0
	71 ± 7	65 ± 12	33 ± 8	57 ± 14				
	62–81	49–78	23–41	31–74				
average SG (*n* = 6)	1.028	1.024	1.026	1.018	1.026	1.018	1.017	1.018

a Concentrations were below an LOD of 0.2 nM in two out of six and three out of six urine samples in volunteers 5 (V5) and 6 (V6), respectively.

b V1 had a higher dietary Se intake due to a considerably higher fish consumption.

c Concentrations in “TMSe non-producers” (V5–V8) were below an LOD of 1.0 nM.

d The table shows the geometric mean (first row), mean ± SD (second row), and range (third row). All concentrations were adjusted to urine specific gravity (SG).

Additional experiments involving dietary intervention showed that ADMS urinary excretion indeed responded to an increased garlic consumption. Volunteer 1, who is a TMSe producer, followed a diet with significantly elevated garlic consumption (10–30 g garlic/week) over several weeks after initial sample collection, and urine collection from this volunteer was repeated. ADMS levels in urine increased from 0.02–0.1 to 1.0– 5.0 μM, and this observation was consistent in all urine samples collected over 2 weeks (*n* = 5). Similarly, urinary levels of ADMS in volunteer 5, who is a TMSe non-producer, increased from 1–2 to 5–10 nM following an increase in garlic consumption (5–10 g garlic/week).

**Figure 5:**
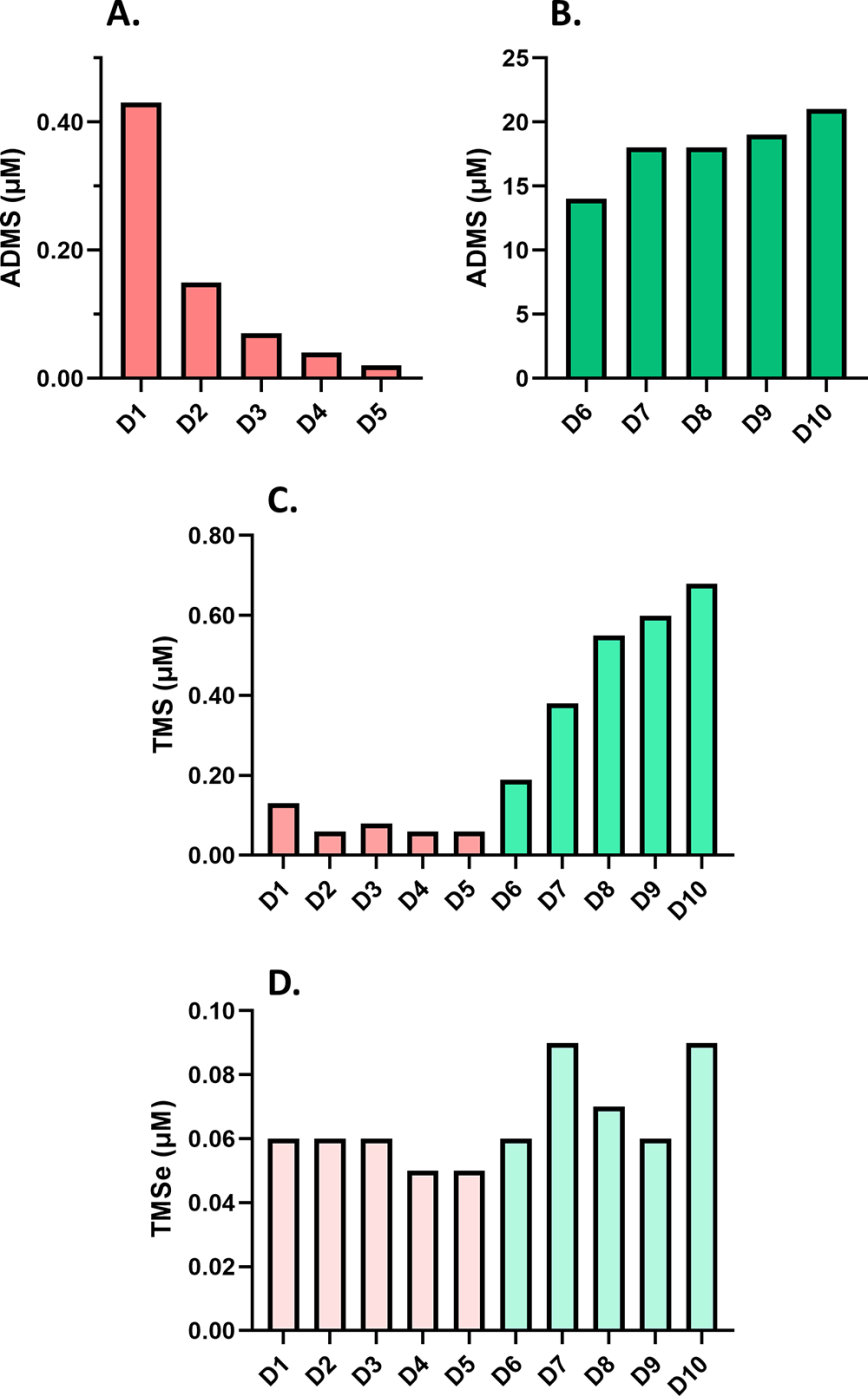
Urinary excretion of products of the INMT enzyme in a garlic supplementation trial. The urinary concentrations of ADMS (A, B), TMS (C), and TMSe (D) are shown in morning (first-pass) urine over five consecutive days of *Allium*-free diet (D1–D5) followed by five consecutive days of daily consumption of ca. 20 g of freshly minced garlic. Note that the general dietary habits of the subject of this experiment (volunteer 1) involve unusually high and regular garlic consumption (10–30 g/week).

Furthermore, after volunteer 1 followed a diet with elevated garlic consumption (10–30 g garlic/week), an *Allium* food was eliminated from the diet for five consecutive days before a controlled daily consumption of ca. 20 g of freshly minced garlic for five consecutive days. Morning (first-pass) urine was collected, and a clear decrease in ADMS urinary excretion over the cessation period was observed, followed by a remarkable increase (up to 1000-fold) in ADMS excretion during the supplementation phase (Figure 5). TMS also displayed a response to elevated garlic consumption (Figure 5), which is explained by the presence of dimethyl thiosulfinate in garlic, which gives rise to dimethyl sulfide, previously reported to be excreted via breath following garlic consumption.^23^ The much higher response in ADMS than that in TMS urinary excretion is in line with the fact that 96–98% of total thiosulfinates are allylic, whereas dimethyl thiosulfinate contributes by only 1–2% of total thiosulfinate in garlic.^23^ It is worth noting that a part of the elevation in urinary TMS may also result from the H_2_S-releasing capacity of organosulfur compounds in garlic to which the health effects of garlic have been linked.^24^ In other words, H_2_S released *in vivo* following garlic consumption may be methylated to dimethyl sulfide by the human body, followed by methylation to TMS by the INMT enzyme. Therefore, the elevation of TMS may support the H_2_S-releasing capacity of garlic, which has not been directly proven in humans.

**Figure 6:**
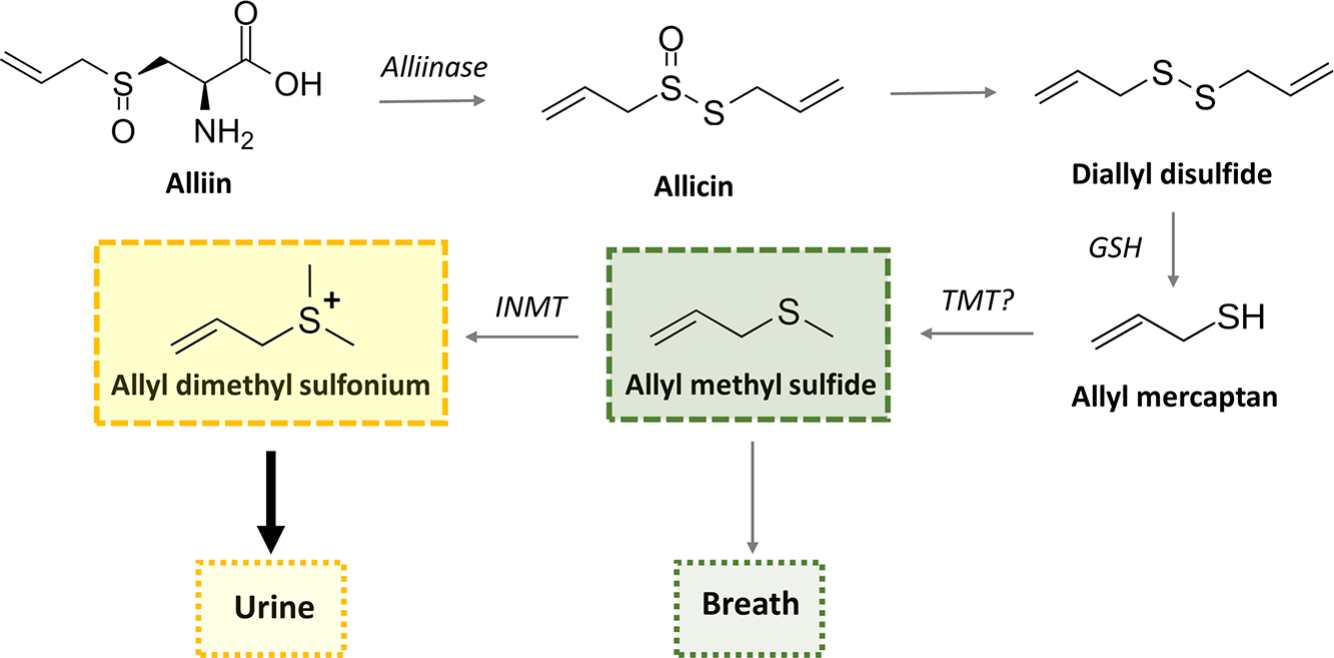
Novel pathway of urinary excretion of allicin-derived organosulfur compounds. Allyl methyl sulfide (AMS) is a central odorous metabolite that is excreted via breath. The enzyme indole ethylamine methyltransferase (INMT) can methylate thioethers including AMS to their respective ionized sulfonium derivatives, which are more hydrophilic and therefore amenable to urinary excretion. Abbreviations: TMT, thiol *S*-methyl transferase; GSH, glutathione; INMT, indoleethylamine *N*-methylransferase (also referred to as thioether *S*-methyltransferase).

*Allium* species, including garlic, contain a variety of sulfur compounds,^25^ including thioethers, which can be targets for the *S*-methylation activity of INMT. However, diallyl disulfide (DADS) is a major component of garlic and primarily arises from degradation of allicin, which is present in garlic at high concentrations (0.3–0.5% w/w).^6^ The reduction of DADS yields allyl mercaptan, which can be in the first step methylated to the volatile and odorous thioether metabolite allyl methyl sulfide (AMS). The enzyme catalyzing this methylation is currently unknown but possibly involves the thiol *S*-methyltransferase (TMT) activity of the METTL7B enzyme, which was recently found to be responsible for the methylation of methylthiol and H_2_S.^26^ In a second methylation step, resulting thioethers can be further methylated to their respective sulfonium compounds by INMT, which leads to permanent ionization, rendering the products of *S*-methylation activity of INMT, including the identified ADMS, much more water-soluble and amenable to excretion via urine (Figure 6). Indeed, the INMT enzyme is primarily expressed in the lungs,^27^ and it is therefore reasonable to suggest that the main driving force for the evolution of the gene for this enzyme is to enable detoxification of volatile sulfur species and excretion in urine.

Apart from the urinary excretion of low concentrations of methylated tryptamine derivatives,^28^ the selenium metabolite TMSe remained the only urinary product of the INMT enzyme reported in humans until recently when we identified the sulfur analogue TMS and highlighted its potential role as a detoxification product of hydrogen sulfide following successive methylation to dimethyl sulfide as well as a biomarker for endogenously produced hydrogen sulfide, which is referred to as the “third gaseous signaling molecule”.^29,30^

The bioactivities of AMS and its precursors have been investigated through *in vitro* tests, which showed effects on cancer cell proliferation^31,32^ as well as immunomodulation,^33^ supporting the therapeutic potential of garlic as a medicinal plant that has been known to humans for thousands of years. The methylation of AMS to the excretory metabolite ADMS can influence the bioavailability of *Allium*-derived bioactive sulfur compounds, and furthermore, the large interindividual variability due to *INMT* polymorphisms suggests interindividual variability in the medicinal and nutritional effects of these plant species, which has been unknown and unaccounted for in the large number of previous epidemiological studies involving the effects of garlic consumption on human health.^34,35^

A sensitive, selective, and long-term biomarker for garlic consumption would open new avenues for such epidemiological investigations since it is difficult to accurately quantify dietary garlic consumption based solely on descriptive data. Although other biomarkers of *Allium* consumption were suggested such as *S*-allyl cysteine and *S*-allyl mercapturic acid,^36^ the advantage of ADMS is that it originates directly from the hydrophobic bioactive components of garlic (e.g., DADS and DATS), which are lipophilic and can therefore accumulate in the human body, and therefore ADMS can be a long-term exposure biomarker to *Allium* food. Indeed, this may explain the consistent detection of background ADMS in urine repeatedly collected from all of the studied volunteers over a relatively long period of time (6 weeks) with moderate variation within each volunteer (Figure 4), even in the absence of dietary intervention or supplementation. Furthermore, ADMS is a permanent ion that enables its detection at low levels by LCMS/MS. The limit of detection of the developed method (0.2 nM or 21 ng L^–1^) is well below the levels in >90% of measured urine samples collected from eight volunteers following a standard western diet (i.e., low to moderate garlic consumption) in the absence of dietary intervention. This suggests that ADMS is sufficiently sensitive to reflect standard dietary conditions and is therefore applicable for assessing *Allium* food consumption over a large scale in the general human population. The developed LCMS/MS method enables rapid quantification within 3 min in untreated urine, which enables high sample-throughput.

In conclusion, the *S*-methylation activity of the INMT enzyme is an unnoticed novel pathway for the metabolism of bioactive sulfur compounds related to *Allium* consumption. ADMS is a major metabolite in this pathway, and the observed interindividual variability in its urinary excretion adds to the significance of this pathway with regard to potentially varying effects of the consumption of these medicinal plants on human health. ADMS can be used in future epidemiological studies investigating the health effects of *Allium* food, serving as a more reliable and more quantitative indicator of *Allium* consumption than food questionnaires. Future studies will involve a systematic investigation of the pharmacokinetic response of ADMS to dietary intervention and supplementation and evaluate the applicability of this novel metabolite as a nutritional biomarker. Finally, the methylation of hydrophobic and bioaccumulating thioethers to their urinary hydrophilic sulfonium derivatives by INMT appears to be a heavily neglected pathway in food and drug metabolism, and its implications warrant further investigation.

## Supporting Information Available

Detection of allyl dimethyl sulfonium (ADMS) with high-resolution mass spectrometry (PDF)
