# Supplementary material for: Allyl Dimethyl Sulfonium: A Novel Urinary Biomarker of Allium Consumption
Source: J Agric Food Chem. 2025 Apr 2;73(15):9128–35. doi: 10.1021/acs.jafc.5c01077 (PMC12007090; doi:10.1021/acs.jafc.5c01077)
Supplement: Supplementary file 1 — jf5c01077_si_001.pdf [file jf5c01077_si_001.pdf]

Electronic Supporting Information

Allyl dimethyl sulfonium: a novel urinary biomarker of *Allium* consumption

Lorenz Steiner[1], Andrea Raab[2], Joerg Feldmann[2], Walter Goessler[1], Bassam Lajin[1,3] *

[1]Institute of Chemistry, Analytical Chemistry for the Health and Environment, University of Graz, Universitaetsplatz 1, 8010 Graz, Austria

[2]Institute of Chemistry, TESLA, University of Graz, Universitaetsplatz 1, 8010 Graz, Austria

[3]Institute of Chemistry, Analytical Chemistry for the Health and Environment, University of Graz, Universitaetsplatz 1, 8010 Graz, Austria

*Corresponding author email address: bassam.lajin@uni-graz.at

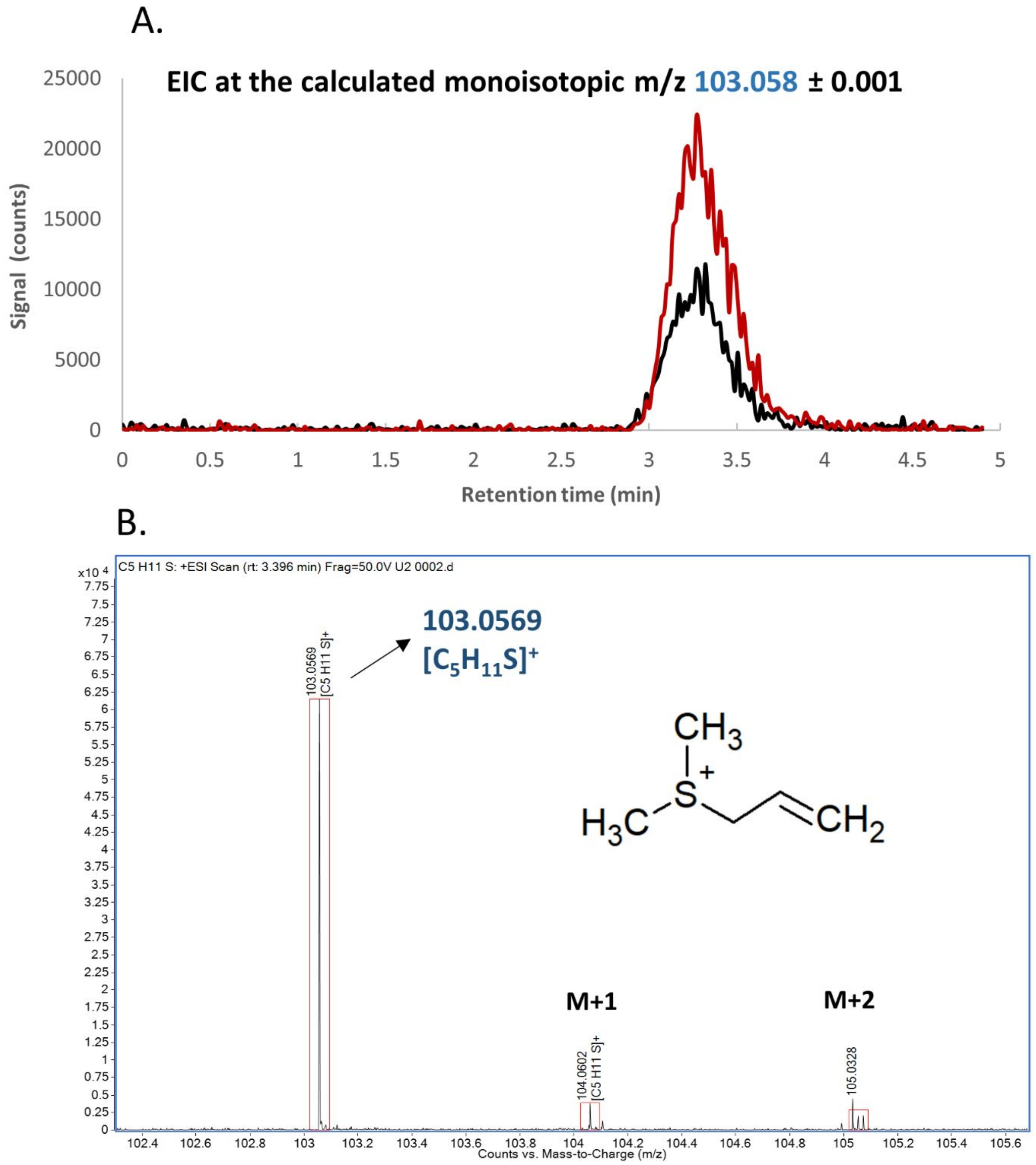

Fig. S1 The detection of allyl dimethyl sulfonium (ADMS) with high resolution mass spectrometry. An LC-QTOF system was employed (Agilent 6546 quadrupole time-of-flight mass spectrometer (Agilent Technologies, Germany). The electrospray ionization source conditions were similar to the Ultivo® tandem MS/MS system (see text). The chromatographic conditions were however modified by replacing the ion-pairing reagent heptafluorobutyric acid with a mobile phase containing 0.2% acetic acid (pH 4.5 adjusted with ammonia) and 20% methanol and using a cation exchange PRP-X200 column (300 X 2.1 mm, Hamilton, USA). The chromatograms (A) were acquired for a urine sample containing 312 nM ADMS before (black) and after (red) spiking with 300 nM pure standard. The measured mass of the detected compound in urine was 103.0569 (B) and the calculated monoisotopic mass of ADMS is 103.0582.
